# Supplementary material for: ETS transcription factors induce a unique UV damage signature that drives recurrent mutagenesis in melanoma
Source: Nat Commun. 2018 Jul 6;9:2626. doi: 10.1038/s41467-018-05064-0 (PMC6035183; doi:10.1038/s41467-018-05064-0)
Supplement: Supplementary file 1 — Supplementary Information [file 41467_2018_5064_MOESM1_ESM.pdf]

Title: ETS transcription factors induce a unique UV damage signature that drives recurrent mutagenesis in melanoma

Mao et al.

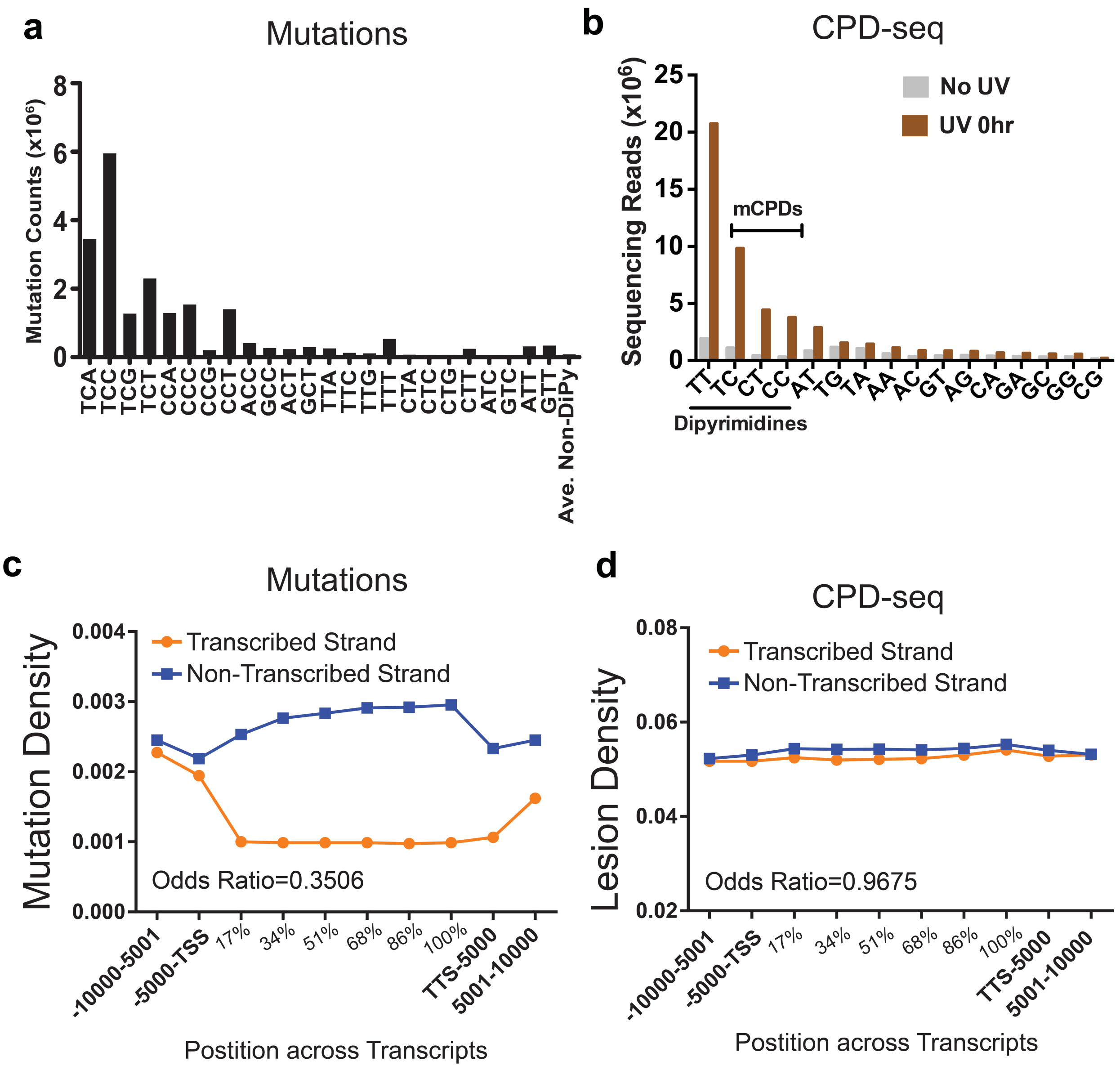

Supplementary Figure 1. CPD-seq reads and UV-induced mutations are enriched in dipyrimidine sequence contexts. **a** All mutations in 184 human melanomas were totaled with respect to the trinucleotide sequence in which they occurred (mutation occurs in the middle base). Complementary sequence contexts were combined and reported such that the mutations altered either C or T nucleotides in the reference genome. Only dipyrimidine containing sequences are shown individually. Mutation counts for non-

dipyrimidine contexts were averaged and shown for comparison. **b** CPD-seq reads associated with dipyrimidine sequences were enriched following UV irradiation of NHF1 cells (UV 0hr). CPD-seq reads from unirradiated NHF1 cells (No UV) were analyzed as a control. The results from one experiment in which the UV 0hr and No UV samples were processed and sequenced in parallel are shown. **c-d** Highly transcribed genes in melanoma (see Methods) were broken into 6 bins per gene, and two additional 5,000bp bins were made before the transcription start site (TSS) and after the transcription termination site (TTS) of each gene, for a total of 10 bins. Mutations (**c**) and CPDs (**d**) were counted in these bins and divided between the transcribed and non-transcribed strands of each gene. While mutations in melanoma tumors show a strong strand bias among highly transcribed genes (Odds ratio = 0.3506 comparing the density of mutations on the transcribed and non-transcribed strand by two-sided Fisher's Exact Test), CPD lesion data for UV 0hr samples had relatively little if any strand bias (Odds ratio = 0.9675).

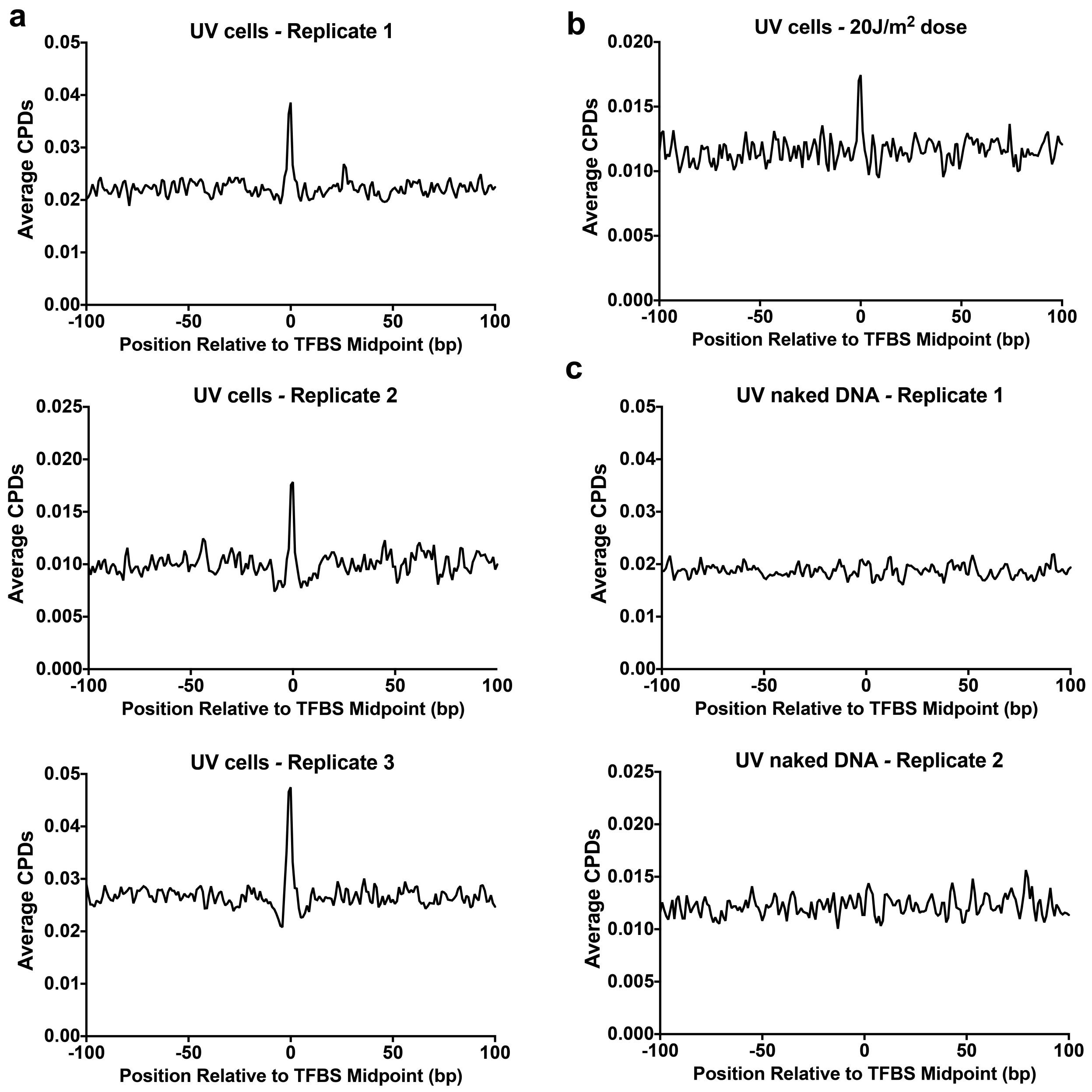

Supplementary Figure 2. UV damage formation is reproducibly elevated at active promoter-proximal TFBS in human cells, but not in naked DNA. **a** Map of CPD lesion formation surrounding active promoter-proximal TFBS (i.e., located in a DNase I hypersensitivity site) from three independent biological replicates. In each case, NHF1 cells were irradiated with 100 J m^{-2} of UV light, and cells were harvested immediately

after UV exposure (0 hr time point), and UV lesions were mapped using CPD-seq. The average density of CPD lesions is plotted surrounding active promoter-proximal TFBS.
b Same as part (**a**), except NHF1 cells were irradiated with a lower UV dose of 20 J m^{-2}.
c Replicate experiments mapping CPD lesions surrounding active promoter-proximal TFBS following UV irradiation (80 J m^{-2}) of isolated genomic DNA (i.e., naked DNA). There was no enrichment of CPD lesions at active TFBS in UV-irradiated naked DNA, indicating that the increase in UV damage formation observed in human cells was not simply due to biases in DNA sequence content at these TFBS.

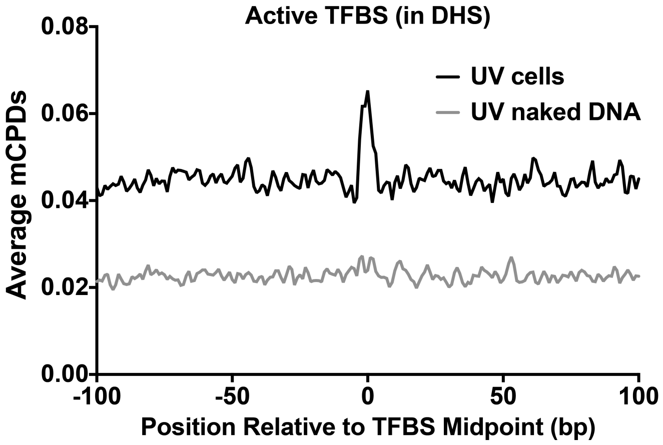

Supplementary Figure 3. Mutagenic CPD (mCPDs) are elevated at active TFBS. Average mCPDs (i.e., CPD lesions at cytosine containing dipyrimidines TC, CT, CC) is plotted surrounding active promoter proximal TFBS. mCPD levels are enriched in UV-irradiated cells (100 J m^{-2}), but not in UV-irradiated naked DNA (80 J m^{-2}).

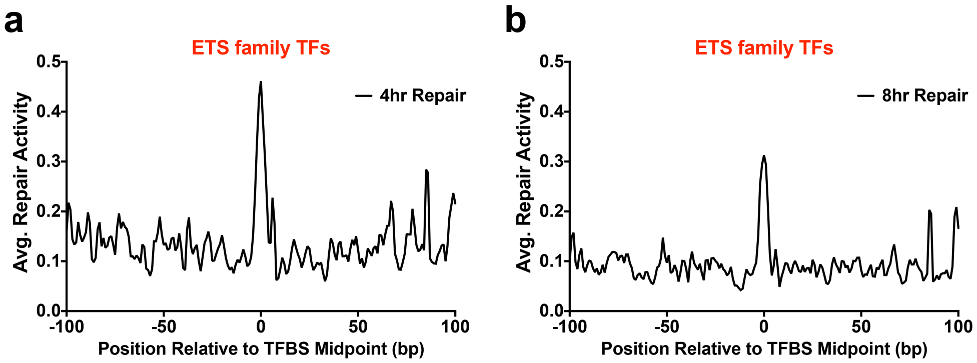

Supplementary Figure 4. CPD repair activity is elevated at ETS family TFBS following UV irradiation of human cells. **a-b** Average CPD repair activity after 4hr and 8hr repair in UV-irradiated NHF1 cells at ETS family TFBS (i.e., ELF1, ELK4, ETS1, and GABPA). CPD repair activity was calculated using the average number of XR-seq reads[1] at locations surrounding active, promoter-proximal ETS family TFBS. XR-seq reads were localized to the putative dipyrimidine lesion associated with each sequencing read.

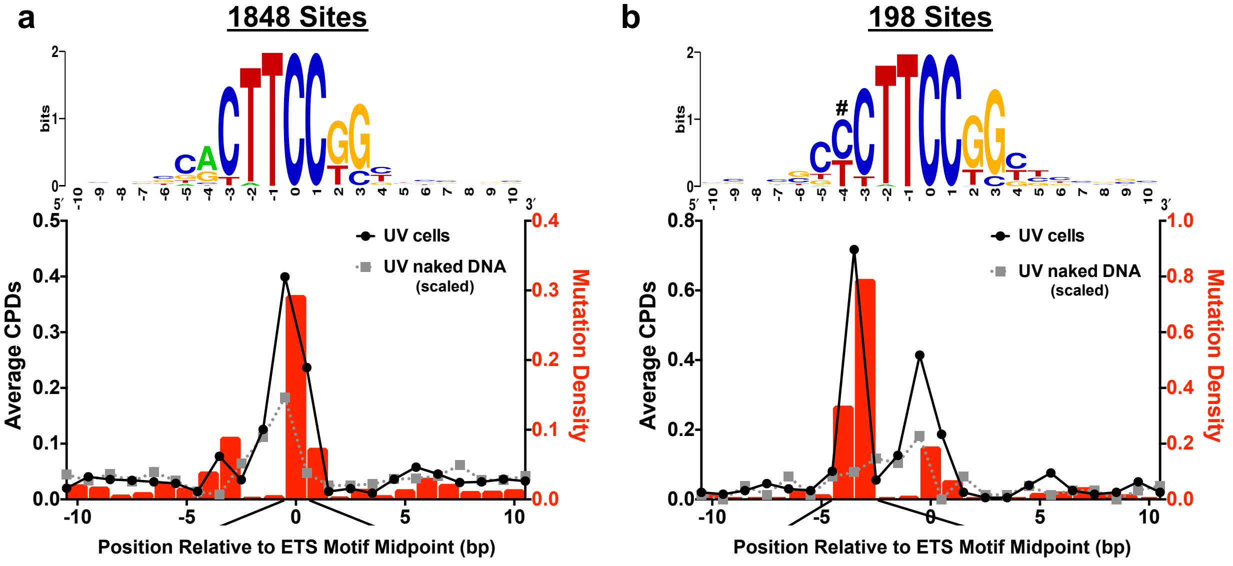

Mutation Count at Midpoint (+0)

TFBS Mutation	Num. of Mutations	Gene	Function
chr19:1479257	9	*C19orf25*	Unknown
chr12:100660920	8	*SCYL2*<br>*DEPDC4*	WNT signaling<br>DEP domain protein
chr19:38865197	8	*PSMD8*	Proteasome non-ATPase
chr9:102861322	8	*ERP44*<br>*INVS**	Protein disulfide isomerase<br>WNT signaling
chr9:132565424	8	*TOR1B*	Torsin family ATPase
chr4:190861982	7	*FRG1*	mRNA binding protein
chr5:179125763	7	*CANX*	Calnexin-family chaperone
chr16:58549393	6	*SETD6**	Histone methyltransferase
chr2:21022857	6	*LDAH**	lipid droplet assoc. hydrolase
chr11:61560113	5	*FEN1**<br>*TMEM258*	Flap endonuclease<br>Transmembrane protein
chr12:49961920	5	*MCRS1**	microspherule protein 1
chr17:45000484	5	*GOSR2*	Golgi trafficking protein
chr5:110074699	5	*SLC25A46*	Mitochondrial solute carrier
chr10:103348032	4	*POLL**<br>*DPCD*	DNA polymerase lambda<br>Unknown
chr12:6862303	4	*MLF2**	Myeloid leukemia factor
chr3:39448199	4	*RPSA**	Laminin receptor
chr4:926179	4	*GAK*<br>*TMEM175*	Cyclin G-associated kinase<br>Transmembrane protein
chr6:31926872	4	*NELFE**<br>*SKIV2L*	Negative Elongation Factor<br>RNA helicase
chr7:2281855	4	*NUDT1**<br>*FTSJ2*	Nudix nucleotide sanitizer<br>SAM-binding protein
:	:	:	:
chr11:111957523	3	*SDHD**<br>*TIMM8B*	Succinate dehydrogenase<br>Mitochondrial import protein
:	:	:	:
chr11:111957541	2	*SDHD**<br>*TIMM8B*	Succinate dehydrogenase<br>Mitochondrial import protein

Mutation Count at Position -4 & -3

TFBS Mutation	Num. of Mutations	Gene	Function
chr6:80714235-80714236	12	*TTK**	Monopolar spindle kinase
chr20:34129795-34129796	11	*ERGIC3**	ER protein
chr19:36870098-36870099	10	*ZFP14*	Zinc finger protein
chr6:57182416-57182417	9	*PRIM2**	DNA primase
chr16:83841525-83841526	8	*HSBP1*	Negative regulator of heat shock factor 1
chr8:125551344-125551345	8	*NDUFB9**<br>*TATDN1*	Mitochondrial Complex I<br>DNase domain
chr1:150266287-150266288	7	*MRPS21*	Mitochondrial ribosomal protein
chr11:61197517-61197518	6	*CPSF7*<br>*SDHAF2**	mRNA processing<br>SDH assembly factor
chr12:14956521-14956522	6	*WBP11*<br>*C12orf60*	mRNA splicing factor?<br>unknown
chr18:3247785-3247786	6	*MYL12A*	myosin light chain
chr1:202927427-202927428	5	*ADIPOR1**	Adiponectin receptor
chr10:98346369-98346370	5	*TM9SF3**	Transmembrane protein
chr17:6554933-6554934	5	*MED31*<br>*C17orf100*	Mediator complex<br>unknown
chr17:45000494-45000495	5	*GOSR2*	Golgi trafficking protein
chr17:57287389-57287390	5	*SMG8*	mRNA degradation
chr5:68665385-68665386	5	*TAF9*<br>*RAD17**	TFIID complex<br>DNA damage signaling
chr6:3259136-3259137	5	*PSMG4*	Proteasome assembly
chr8:124054557-124054558	5	*DERL1**	ER protein degradation
chrX:69353705-69353706	5	*IGBP1**	Protein phosphatase 2A regulator

Supplementary Figure 5. Elevated CPD formation and mutation density coincide at specific locations in non-promoter proximal ETS TFBS**. a** CPD formation and mutation

density in melanoma tumors is elevated at specific locations within 1848 active, non-promoter proximal TFBS for the ETS TFs ELK4, ETS1, and GABPA. TFBS were aligned based on the location and DNA strand of the ETS consensus sequence. Top panel depicts the consensus sequence of the aligned ETS binding sites, which was constructed using the weblogo tool[2]. Middle panel plots the mutation density from 184 melanoma tumors relative to the average CPD levels following UV irradiation in NHF1 cells and isolated DNA *in vitro* (naked DNA). CPD values are plotted at half integer locations, which reflect the average number of CPD lesions forming between the two adjacent nucleotides. CPD enrichment relative to naked DNA was greatest for lesions occurring between positions -4/-3, -1/0, and 0/+1 relative to the ETS motif midpoint. Lower panel lists the most recurrent ETS TFBS mutations among 184 melanoma tumors at position 0 relative to the ETS motif midpoint, and the genes associated with these TFBS. Non-promoter proximal ETS TFBS were frequently located near the 5' end of a gene (e.g., in the 5' untranslated region). TFBS that are associated with genes linked to cancer are indicated with an asterisk (*). **b** Analysis of CPD lesion formation and mutation density at a subset of active, non-promoter proximal ETS TFBS that have a pyrimidine nucleotide at position -4 relative to the ETS motif midpoint (indicated with #), and thus can form CPD lesions between position -4/-3. Top and middle panels are the same as in part **a**, plotted for this TFBS subset. Lower panel lists the most recurrent ETS TFBS mutations at positions -4 and -3 (summed) relative to the ETS motif midpoint. TFBS that are associated with genes linked to cancer indicated with an asterisk (*).

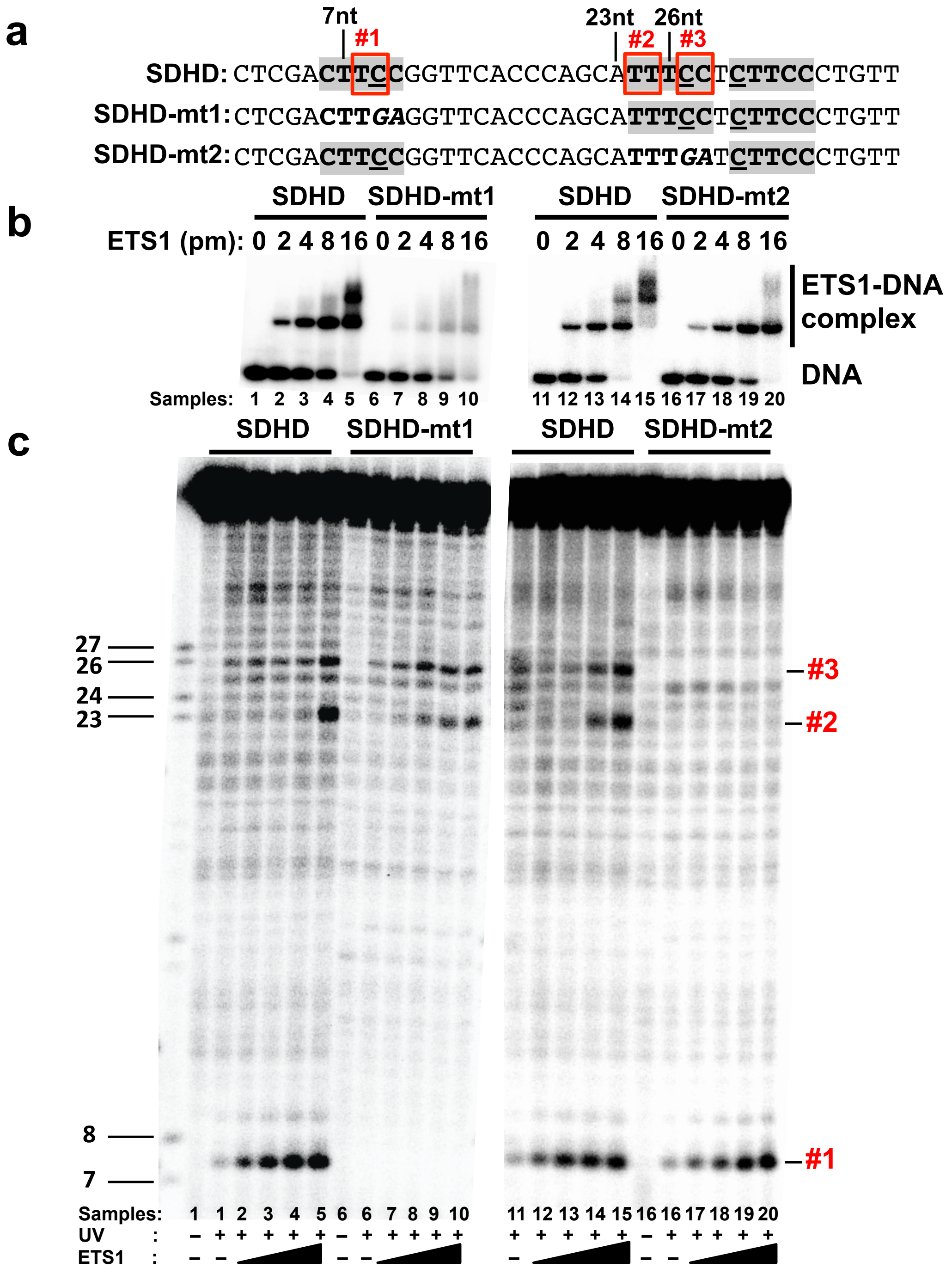

Supplementary Figure 6. Mutations in ETS motifs in the *SDHD* promoter disrupt ETS1 binding and UV damage induction. **a** DNA sequences of wild-type and mutated *SDHD* promoter fragment. Mutant1 (SDHD-mt1) contains point mutations in the consensus

sequence of ETS motif-1 and mutant2 (SDHD-mt2) includes point mutations in ETS motif-2. **b** Gel shift showing binding of purified ETS1 protein to the wild-type *SDHD* promoter DNA fragment, significantly reduced binding to SDHD-mt1, and altered binding pattern to SDHD-mt2. Each reaction has 4 pmol of ^{32}P-labeled DNA. **c** Sequencing gels (15%) showing CPDs in wild-type SDHD, SDHD-mt1, and SDHD-mt2. Synthesized *SDHD* oligos with known lengths are loaded in the first lane and the sizes of CPD bands are determined by comparing to *SDHD* oligos. Numbers shown on the left indicate sizes (nt) of each *SDHD* oligo, and the three CPD ‘hotspots’ (see **a**) are indicated on the right (i.e., #1, #2 and #3).

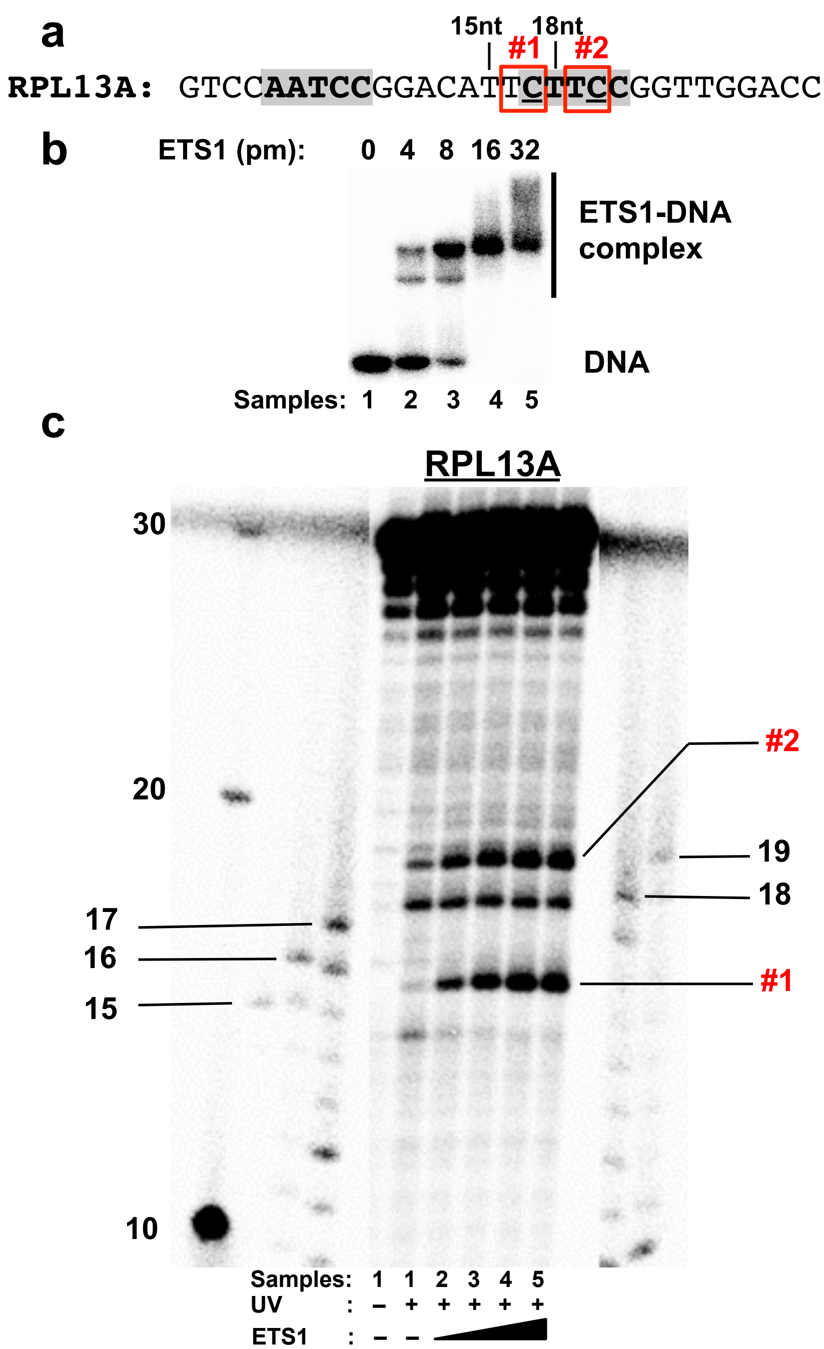

Supplementary Figure 7. Determination of the precise locations of UV-induced CPDs in *RPL13A*. **a** Sequence of *RPL13A* promoter fragment. The two putative ETS motifs are shown in gray background and highlighted in bold. The highly recurrent mutations in

melanoma tumors are underlined. The two CPD ‘hotspots’ are boxed and their distances to the 5’ end are labeled. **b** Gel shift showing binding of purified ETS1 protein to ^{32}P-labeled *RPL13A* promoter fragment. DNA used in each reaction is 4 pmol, and the amount of protein is shown on the top. **c** Sequencing gel showing stimulation of CPDs by ETS1 protein in *RPL13A*. The precise locations of CPDs are determined by comparing the CPD bands to synthesized *RPL13A* oligonucleotides (oligos) with known lengths. Some synthesized oligos show multiple bands, presumably due to the presence of intermediates during oligo synthesis. Only one synthesized oligo was loaded in each size lane, and the top band in the lane was recognized as the real size. Numbers on the left and right of the gel indicate oligo sizes (nt). The two CPD ‘hotspots’ are depicted on the right (i.e., #1 and #2).

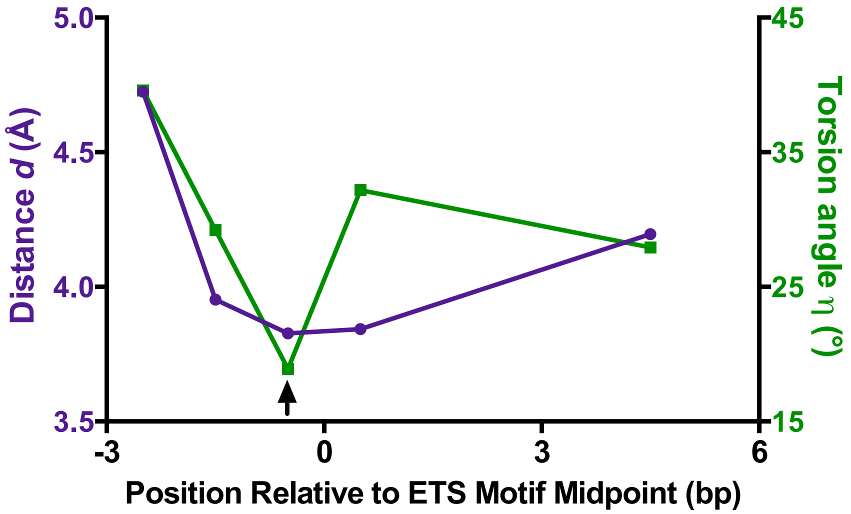

Supplementary Figure 8. Plot of the distance **d** and torsion angle (**η**) between C5-C6 bonds of adjacent pyrimidines in the GABPA-bound DNA structure (PDB ID: 1AWC).

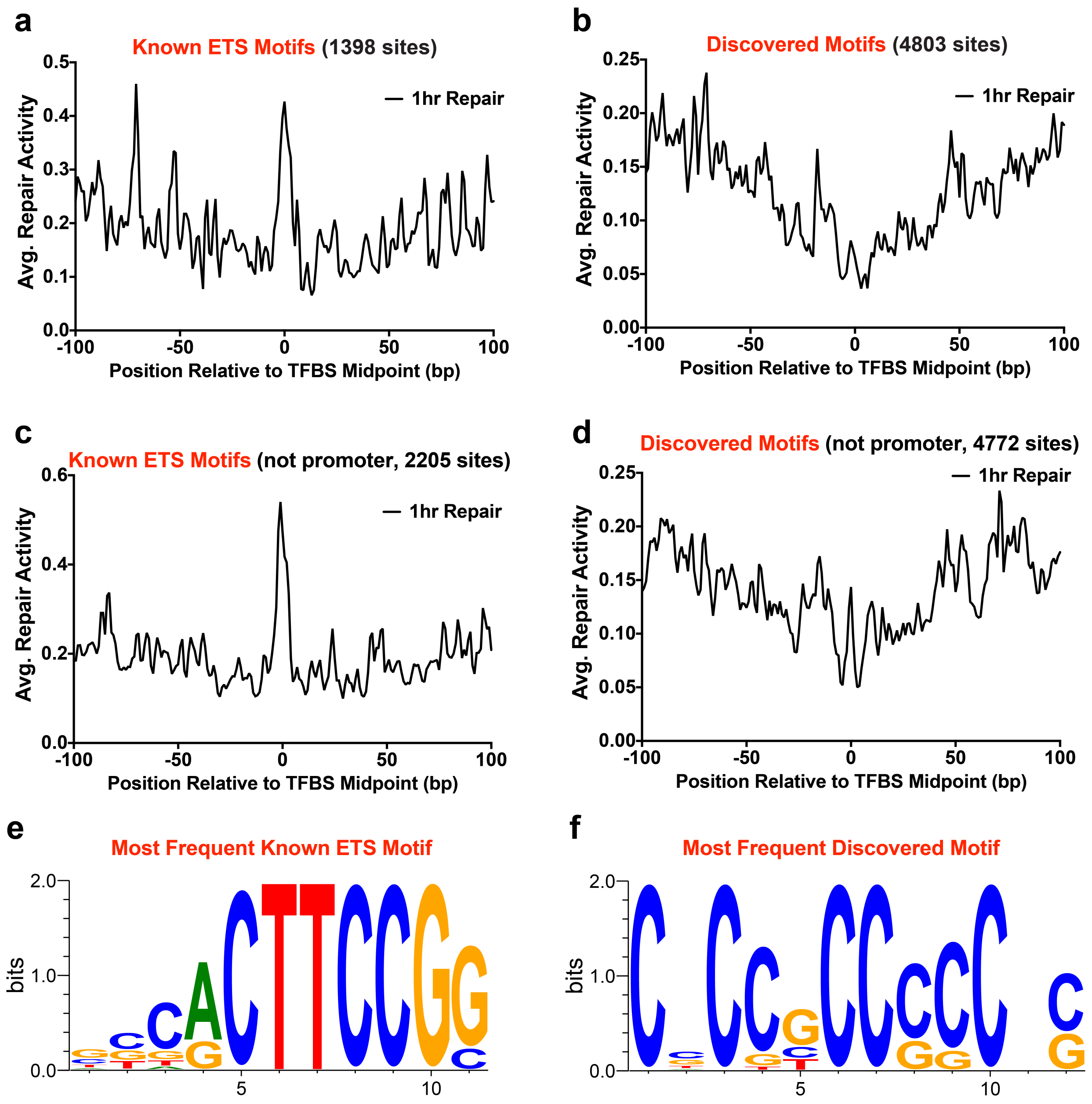

Supplementary Figure 9. High repair activity is associated with known ETS motifs, but not discovered ETS motifs. **a-d** Average CPD repair activity at 1 hr repair in UV-irradiated NHF1 cells at ETS family TFBS (i.e., ELF1, ELK4, ETS1, and GABPA). CPD repair activity was calculated using the average number of XR-seq reads[1] at locations surrounding active, promoter-proximal ETS family TFBS (**a-b**), and active ETS family

TFBS located outside promoters (**c-d**). Panels A and C depict repair activity for binding sites containing known ETS motifs, while panels B and D depict repair activity for discovered ETS motifs. (**e-f**) Sequence logos depicting the consensus sequence of the most frequent known ETS motif (**e**) and most frequent discovered ETS motif (**f**), which were constructed using the weblogo tool[2].

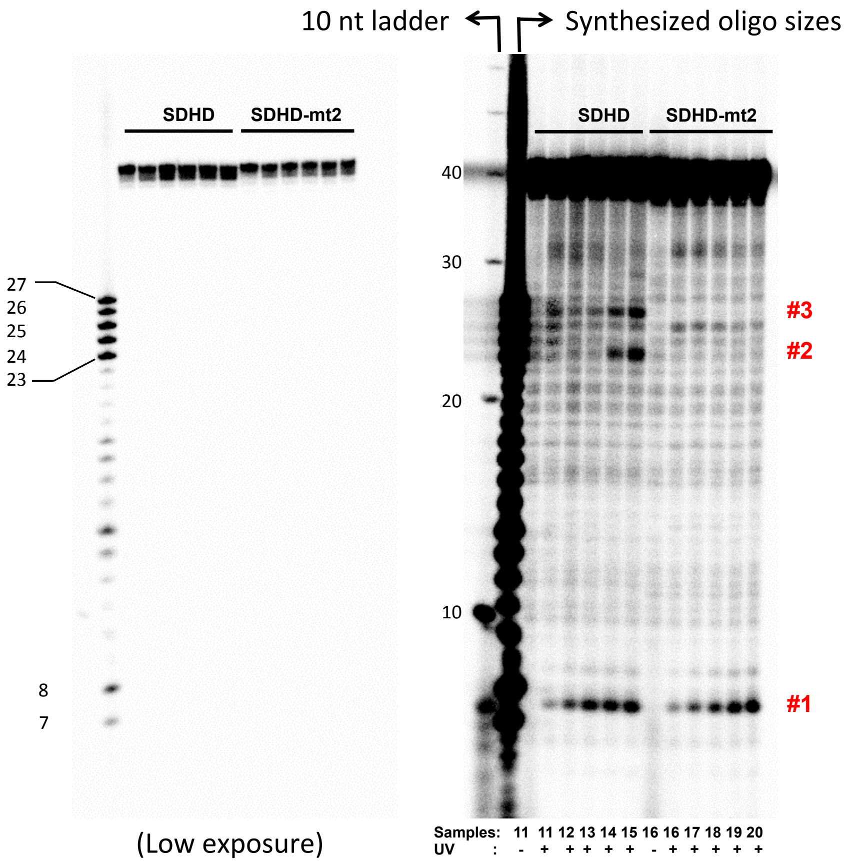

Supplementary Figure 10. Uncropped gel image for gel shown in right panel of Supplementary Figure 6c, showing molecular weight/size markers. Two different exposures are shown.

Supplementary Table 1: Recurrent mutations in melanoma that occur in mutation hotspot at position 0 (TTCC, underline indicates mutation) in active promoter-proximal ETS binding sites

TFBS Mutation	Num. of Mutations#	Gene	Function
chr19:49990691	10	*RPL13A*	GAIT complex subunit
chr3:122135048	9	*WDR5B*	CUL4-DDB1 complex?
chr7:5553439	8	*FBXL18**	F-box protein
chr7:111846581	8	*ZNF277, DOCK4**	Zinc-finger protein, GEF
chr9:132565424	8	*TOR1B*	Torsin family ATPase
chr11:47448145	7	*PSMC3*	Proteasome ATPase
chr16:15982556	7	*FOPNL**	Centrosome function
chr17:72772528	6	*NAT9 TMEM104*	N-acetyltransferase Transmembrane protein
chr17:79935419	6	*ASPSCR1**	Translocations in sarcoma, carcinomas
chr19:16296108	6	*FAM32A**	Assoc. with ovarian tumors
chr19:45004586	6	*ZNF180*	Zinc-finger protein
chr19:46390154	6	*IRF2BP1*	IRF2 co-repressor
chr3:88108326	6	*CGGBP1*	Transcription regulator

#Only the most frequent recurrent mutations at this location in the ETS motif are listed.
*Genes linked to cancer.

Supplementary Table 2: Recurrent mutations in melanoma that occur in mutation hotspot at positions -3 and -4 (relative to motif midpoint) in active promoter-proximal ETS binding sites

TFBS Mutation	Num. of Mutations#	Gene	Function
chr19:49990694-49990695	47	*RPL13A*	GAIT complex
chr3:16306504-16306505	32	*DPH3*<br>*OXNAD1*	Dipthamide biosyn.<br>Oxidoreductase
chr3:101280670-101280671	29	*TRMT10C*	RNase P subunit
chr11:47448148-47448149	23	*PSMC3*	Proteasome ATPase
chr1:179846984-179846985	21	*TOR1AIP2*<br>*TOR1AIP1*	Torsin A cofactor<br>Torsin A cofactor
chr6:149867285-149867286	18	*PPIL4*	Prolyl isomerase
chr1:100598552-100598553	16	*SASS6**<br>*TRMT13*	Centriole component<br>tRNA methylase
chr12:498775-498776	15	*CCDC77*<br>*KDM5A**	Coiled-coil protein<br>Histone demethylase
chr14:53173817-53173818	12	*PSMC6*	Proteasome ATPase
chr1:19923392-19923393	11	*MINOS1*	Mitochondrial inner membrane organizer
chr11:61735191-61735192	10	*FTH1*	Ferritin heavy subunit
chr2:68479926-68479927	10	*PPP3R1*	Calcineurin subunit
chr20:32581032-32581033	10	*RALY*	hnRNP
chr22:31556120-31556121	10	*RNF185*	Ring finger protein
chr5:133702909-133702910	10	*CDKL3**<br>*UBE2B*	Cyclin-dep. kinase<br>Rad6 homolog

#Only the most frequent recurrent mutations at this location in the ETS motif are listed.
*Genes linked to cancer.

Supplementary Table 3: Expression of ETS transcription factors in melanocytes

Name	ETS Subfamily	Expression in Melanocytes (RPKM)*
**ETS1**	Class I - ETS	11.1
**ETV5**	Class I - PEA3	6.7
**ELF1**	Class II - ELF	3.2
**ERF**	Class I - ERF	2.1
**ETV3**	Class I - ERF	1.7
**ETV1**	Class I - PEA3	1.7
**ELF2**	Class II - ELF	1.5
**ELK4**	Class I - TCF	1.4
**ELK1**	Class I - TCF	1.3
**ETV6**	Class II - TEL	1.2
**ETV4**	Class I - PEA3	1
**ELK3**	Class I - TCF	0.9
**FLI1**	Class I - ERG	0.7
**GABPA**	Class I - GABPA	0.7
**ETS2**	Class I - ETS	0.4
**ELF4**	Class II - ELF	0.3
**ETV7**	Class II - TEL	0.3

*RNA-seq data from ENCODE. ETS transcription factors with an expression <0.1 RPKM in melanocytes are not listed.

Supplementary References

1. Adar, S., Hu, J., Lieb, J.D. & Sancar, A. Genome-wide kinetics of DNA excision repair in relation to chromatin state and mutagenesis. *Proc Natl Acad Sci U S A* **113**, E2124-33 (2016).
2. Crooks, G.E., Hon, G., Chandonia, J.M. & Brenner, S.E. WebLogo: a sequence logo generator. *Genome Res* **14**, 1188-90 (2004).
